# Understanding the Association Between Humor and Emotional Distress: The Role of Light and Dark Humor in Predicting Depression, Anxiety, and Stress

**DOI:** 10.5964/ejop.10013

**Published:** 2023-11-30

**Authors:** Alberto Dionigi, Mirko Duradoni, Laura Vagnoli

**Affiliations:** 1 Studio Psi.Co., Cattolica, Italy; 2 Department of Education, Languages, Interculture, Literatures, and Psychology, University of Florence, Florence, Italy; 3 Meyer Children’s Hospital IRCCS, Pediatric Psychology, Florence, Italy; Università Cattolica del Sacro Cuore, Milan, Italy

**Keywords:** comic styles, humor, depression, anxiety, stress

## Abstract

Despite increasing interest in the relationship between humor and psychological distress, investigations have failed to focus on specific categories of humor and negative mental conditions. A sample of 686 Italian participants (187 men and 499 women), aged between 20 and 76 years, completed an online survey, data from which was used to investigate the relationship between eight comic styles, depression, anxiety, and stress. Findings from the multiple linear regression demonstrate benign humor as a protective factor of all three variables considered, while irony was positively associated with anxiety and stress. Wit was a protective factor associated with anxiety, while sarcasm was positively related to depression. No significant correlations emerged between the other variables considered. These findings highlight how specific categories are linked to varying dimensions of emotional distress, which are discussed with reference to the extant literature.

There has been an increased interest in the significant role humor plays in the lives of individuals, particularly its contribution to the promotion of mental health over the past 40 years; several studies in this context have focused on the relationship between humor and well-being (Martin, 2019; Ruch & McGhee, 2014). Humor allows people to distance themselves from problems, thereby increasing positive emotions and easing tension—it operates as an effective coping strategy to be adopted in the face of stress (Crawford, & Caltabiano, 2011). Despite the universality of humor, it remains a complex and multifaceted phenomenon, occurring as an amalgamation of emotional, cognitive, and behavioral components (Martin & Ford, 2018; Ruch, 2008). Humor has the potential to contribute to the well-being of individuals, through certain components such as; a) a cognitive, a stimulus that a person perceives as funny; b) an emotional response, which leads to mirth; and c) a physiological response, such as a laugh or smile (Martin & Ford, 2018; Wellenzohn et al., 2016).

According to recent evidence, while certain forms of humor are psychologically positive and adaptive, the expression of other forms of humor represents less desirable and less healthy modes of interaction (Dozois et al., 2009). In this study, we aimed at investigating how specific categories of humor (namely the comic styles) relate to depression, anxiety, and stress. The relations between humor and psychological well-being have been analyzed by various authors, most frequently with the support of the Humor Styles Questionnaire (HSQ) (Martin et al., 2003). The associated framework indicates four humor styles, two of which are positive (affiliative, which involves the use of jokes and friendly banter to facilitate interpersonal bonds; and self-enhancing, which involves humor as a coping strategy to deal with life stressors), and two that are negative (aggressive, which involves the teasing and ridiculing of others to enhance the self; and self-defeating, which involves excessively self-disparaging humor to amuse others at one’s own expense to ingratiate oneself or gain approval). Generally, the two adaptive humor styles are positively associated with extraversion, agreeableness, conscientiousness, openness, and well-being, while they are negatively correlated with neuroticism. Conversely, the two negative styles demonstrate a negative association with agreeableness and conscientiousness, while they are positively associated with neuroticism and poor well-being (Fritz, 2020; Martin et al., 2003; Plessen et al., 2020; Vernon et al., 2008). However, the HSQ uses a theoretical model of humor at a general level, which is based on the description of humor and how and when it is used. In this conceptualization, humor mainly reflects two functions represented by enhancing oneself and enhancing relationships with others. People can accomplish these functions in a adaptive way, using affiliative and self-enhancing humor or maladaptively, using aggressive and self-defeating humor. To further the efficiency of detailed investigations regarding the nature of humor, Comic Style Markers (CSM), which focuses on a list of eight lower-level styles, was developed recently (Heintz, 2023; Ruch, Heintz, et al., 2018). The eight comic styles were established to identify specific categories of humor and to describe individual differences in their use. Research shows that these styles reflect established categories of humor (in the broad sense) and that they are narrower than the ones in the Humorous Behavior Q-Sort Deck (HBQD) and HSQ, which allows for a more fine-grained differentiation of humor-related styles (Heintz & Ruch, 2019).

The eight comic styles can be differentiated as lighter or darker styles of humor, which collectively include fun, humor, nonsense, wit, irony, satire, sarcasm, and cynicism (Ruch, Heintz, et al., 2018). The four lighter styles, which relate to benign and social affect, behaviors, cognitions, and goals are; (1) *fun*, aimed at spreading good mood and good companionship; (2) *humor,* aimed at arousing sympathy towards the shortcomings of a fellow human, discovering discrepancies in everyday experiences, and treating them in a humorous and benevolent manner; (3) *nonsense,* based on experimentation with incongruities and ridiculousness with no specific purpose; and (4) *wit*, which relates to the ability to create clever connections between ideas and thoughts. Conversely, the darker styles that lack this benevolent affect are mostly based on mockery and ridicule. These include; (1) *irony,* reflecting a contrast or incongruity between expectations for a situation and reality, characterizing the opposite of the expressed meaning; (2) *satir*e, directed at criticizing and correcting shortcomings, misconduct, and moral wrongdoings with the intent to improve the world; (3) *sarcasm*, grounded on the need to be critical of others and convey contempt; and (4) *cynicism,* aimed at devaluing commonly recognized values (see Appendix). Previous research that compared the Humor Styles and the Comic Styles showed that three of the 12 styles overlap (i.e., fun/affiliative, benevolent humor/self-enhancing, sarcasm/aggressive) (Heintz & Ruch, 2019). However, this do not mean that the styles are interchangeable as they are based on detecting different aspects: generally, the comic styles are more sophisticated than the humor styles.

Previous studies revealed that the association between humor and emotional distress were conducted only using the Humor Styles Model showing how each humor style is differently related to emotional distress and specific associations with anxiety, stress, and depression emerged (Martin & Ford, 2018; Schneider et al., 2018). To this end, the current study documents our investigation of how benign and malicious categories of humor, as defined by the Comic Style Markers, relate to depression, anxiety, and stress.

## Humor, Depression, Anxiety, and Stress

Multiple studies have previously investigated the relationship between humor and negative psychological states such as depression, anxiety, and stress. In this context, while benign humor, aimed at amusing others and pointing out the funny side of adversities in a good-natured manner, was found to yield better regulation of negative emotions, malicious, or “dark” humor, based on injurious, mean-spirited goals and attitudes has been positively associated with negative emotions (Papousek et al., 2017). The following subsections examine the relationship between humor, depression, anxiety, and stress, with a brief review of salient points put forward by existing literature.

### Humor and Depression

Humor and depression are capable of maintaining a mutually influential relationship. For example, each humor style has a unique effect on the quality of one’s social relationships, likeability, and attractiveness ratings. According to research, depressive symptoms are moderately negatively correlated with affiliative and self-enhancing forms of humor, while positively correlated with the use of self-defeating humor (Frewen et al., 2008; Martin et al., 2003). Moreover, the appearance of depressive symptoms was correlated with the perception of support from one’s social circle, which has been associated with the use of specific humor styles (Dyck & Holtzman, 2013). Many studies showed positive correlations between depressive symptoms and aggressive and self-defeating humor styles while negative correlations were observed between depression and self-enhancing uses of humor (Rnic et al., 2016; Schermer et al., 2017). Specifically, as depression as to do with a negative view of self, it is reasonable to find a positive relationship with self-defeating humor that is mainly directed to hurting oneself (Kfrerer et al., 2019). In a recent meta-analysis, humor interventions were reported to significantly reduce depression and anxiety levels among adults, with an increase in the quality of sleep (Zhao et al., 2019). To date, no studies were conducted to investigate the relationship between depression and the eight comic styles, but certain studies have examined the relationship between the latter and general and subjective well-being, which can represent an indicator of negative affect: experiencing a loss of satisfaction, happiness and/or psychological well-being may lead to clinical depression (Cummins & Lau, 2006). Positive affectivity was positively correlated to the lighter styles and negatively with cynicism; negative affectivity demonstrated positive relations with sarcasm and cynicism and negative associations with benevolent humor (Ruch, Wagner, & Heintz., 2018). In another study, humor and fun were positively related to psychological well-being, while no significant negative predictors were detected (Dionigi et al., 2021). This result is similar to previous research that has shown controversial findings between aggressive humor and depression: for example, some studies found no significant relationship between them (Besser et al., 2011; Martin et al., 2003).

### Humor and Anxiety

Humor, as an effective strategy for reducing non-clinical and clinical anxiety (Abel, 2002; Ford et al., 2012), is mediated by various functions in its relationship with anxiety, such as emotion regulation, distraction, and reframing strategies for stressful events (Kuiper et al., 2014). The use of benign humor is considered a powerful coping strategy as it can facilitate the reinterpretations of the subjective meaning of an emotionally negative event, thereby altering its emotional impact (Perchtold et al., 2019). According to existing studies, individuals using positive sense of humor are less prone to developing anxiety and stress and are less prone to perceive negatively stressful life events, relative to those with a lower sense of humor (Kuiper, 2012). Research illustrates the differential relationship of positive and negative styles of humor with the prediction of anxiety levels. Benevolent humor styles, such as affiliative humor and self-defeating humor, were linked to low anxiety (Kuiper et al., 2004; Martin et al., 2003) and negatively associated with social anxiety (Tucker et al., 2013). Self-defeating humor was found to be related to higher levels of anxiety in some studies (Kuiper et al., 2004; Martin et al., 2003; Schneider et al., 2018), while there was a lack of correlation in others (Ford et al., 2017). Aggressive humor was generally unrelated to anxiety level (Schneider et al., 2018).

When considering specific categories of humor through the lens offered by the Comic Style Markers, research demonstrates that individuals with greater habitual use of benevolent humor and lesser habitual use of dark humor were found to be more involved in relatively more significant use of these particular reappraisal strategies (Perchtold et al., 2019). More recently, a significant negative relationship was determined between worry (the cognitive component of anxiety) and the two benevolent styles of fun and humor, while a positive relationship emerged between cynicism and worry (Dionigi et al., 2021).

### Humor and Stress

Humor represents a protective factor that helps to cope with stress through its role as a moderator between negative life events and mood disturbance (Martin & Ford, 2018). Individuals who use humor as a coping strategy are more likely to approach stressful situations using a relatively positive perspective, engaging in cognitive and behavioral strategies more often, and thereby experiencing relatively lower stress (Kuiper &. Harris, 2009). Moreover, the use of humor has been evaluated as an adaptive emotion regulation strategy (Kugler & Kuhbandner, 2015; Samson & Gross, 2012). In this regard, self-defeating humor showed positive relationships with increasing stress levels, meanwhile individuals using affiliative humor reported lower level of stress (Cann & Collette, 2014; Putz & Breuer, 2017). Humor is employed both as a cognitive and behavioral strategy to deal with stress. While humor physiologically decreases the level of pro-stress factors, it improves mood-elevating anti-stress factors, thereby resulting in a reduction of stress responses (Bennett & Lengacher, 2009). In a recent study (Fritz et al., 2017), humor was found to have stress-buffering effects and a negative relationship with stress in a sample of patients with diagnosed fibromyalgia syndrome.

## Aim of the Study

The present study was aimed at investigating how specific categories of humor relate to depression, anxiety, and stress. Considering extant research focused on this subject (Dionigi et al., 2021; Perchtold et al., 2019; Ruch, Heintz, et al., 2018), the specific hypotheses of this cross-sectional study are as follows:

H1. Benevolent humor, which represents the form of benign humor (correlated to coping humor), is expected to share a relationship with lower depression, anxiety and stress. Fun, Wit and Nonsense, will be tested exploratively.

H2. The Mockery styles, particularly Cynicism, would demonstrate a positive association with depression, anxiety, and stress due to their positive influence on emotional distress. A similar pattern is expected for irony due to its ambivalent content. Satire and Sarcasm will be tested exploratively.

## Method

### Participants

Data were collected from 686 Italian participants (187 men and 499 women), aged between 20 and 76 years (*M* = 42.88 years; *SD* = 12.53). Participants were well-educated adults (4.2% of the sample had a lower secondary school diploma, 40.1% had an upper secondary school diploma, 31.9% had a university degree, 17.2% had a master’s degree, and 6.6% had a doctorate). In terms of marital status, 231 (33.7%) were unmarried, 371 (54.1%) were married or cohabiting, 79 (11.5%) were divorced, and five (0.7%) were widowed.

### Measures

The *Comic Style Markers* (CSM) (Ruch, Heintz, et al., 2018) is a self-report questionnaire wherein participants rate the extent to which 48 statements apply to their typical expressions of humor on a 7-point Likert scale, ranging from 1 (strongly disagree) to 7 (strongly agree). Total scores correspond to the mean of the six items, with higher scores corresponding to higher use of a specific comic style. This study adopted the Italian version of the questionnaire (Dionigi et al., 2022). The eight scales demonstrated good to acceptable reliabilities (McDonald’s ω: fun = .84; humor = .72; nonsense = .86; wit = .82; irony = .78; satire = .78; sarcasm = .72; cynicism = .82).

The *Depression Anxiety Stress Scales-21* (DASS-21) (Lovibond & Lovibond, 1995) is a self-reporting questionnaire with a total of 21 items distributed across three subscales, each of which is based on a four-point rating scale to measure varying categories of emotional distress (depression, anxiety, and stress). The severity of the emotional distress increases with the score. The current study adopted the Italian version of the scale (Bottesi et al., 2015). The three subscales in this study were demonstrative of optimal reliabilities (McDonald’s ω: depression = .90; anxiety = .86; stress = .88).

### Procedure

Data were collected utilizing an online survey (i.e., Google Forms web-link). The link to the survey was posted on social media and sent using mailing lists. The research design was cross-sectional, and the inclusion criteria were: a) individuals aged 18 years or older; and b) being an Italian citizen. The survey also contained an explanation of the aim of the study and consent to participate. The study was performed in accordance with the local ethical guidelines, and all participants were guaranteed anonymity.

### Statistical Analysis

All data were analyzed using SPSS 25.0 (IBM). First, a power analysis was performed to determine the adequate sample size, based on the needs of the analysis type. This was accomplished using the G*Power software (Faul et al., 2007, 2009). The correlations among CSM and DASS-21 were analyzed using Pearson correlation coefficients. For Pearson’s correlation, a sample size of *N* = 616 would be required to achieve a statistical power of .80, while being able to capture even a small effect size (*r* = .10) and assuming a significance level of .05. Moreover, as our study is primarily based on correlation, we accounted for the required sample size to achieve a stable measurement-error-free correlation. In the context of this study (i.e., population correlation ρ = .10; composite score reliability derived from other works ω = .70–.80), a stable measurement-error-free correlation would be met at *N* = 380–510 (Kretzschmar & Gignac, 2019). Measurement-error-free correlation is a condition where estimate deviations should fluctuate only within a predefined “corridor of stability” that should have upper and lower bounds equal to |0.10| (Schönbrodt & Perugini, 2013).

For linear hierarchical regression analysis, however, a sample size of *N* = 213 individuals would be sufficient to ensure a statistical power of .90, assuming a very small effect size (*f*^2^ = .05) and a significance level of .05. Thus, the sample size of *N* = 686 participants was deemed adequate for the purposes of this study. Eventually, for each Pearson correlation, we assessed the variable normality (asymmetry and kurtosis values), homoscedasticity, and linearity. Hierarchical regression of the comic styles with anxiety, stress, and depression was conducted following the testing for potential multicollinearity issues.

## Results

As a first step, descriptive statistics of the collected variables were generated and presented for the entire sample and disaggregated by gender (Table 1).

**Table 1:** Descriptive Statistics of the Collected Variables

	*M (SD)*		
Variable	Total	Females	Males
1. Age	42.88 (12.53)	41.95 (12.16)	45.35 (13.19)
2. Gender	72.7% (females), 27.3% (males)	—	—
3. Depression	4.81 (4.24)	4.84 (4.19)	4.73 (4.38)
4. Anxiety	6.47 (3.27)	6.68 (3.39)	5.90 (2.87)
5. Stress	7.17 (4.21)	7.26 (4.22)	6.90 (4.18)
6. Fun	4.27 (1.25)	4.19 (1.24)	4.50 (1.26)
7. Humor	5.24 (0.86)	5.20 (0.87)	5.36 (0.85)
8. Nonsense	4.80 (1.25)	4.68 (1.22)	5.13 (1.28)
9. Wit	4.87 (0.98)	4.81 (0.98)	5.03 (0.96)
10. Irony	4.23 (1.11)	4.17 (1.10)	4.41 (1.12)
11. Satire	4.44 (1.09)	4.32 (1.08)	4.76 (1.06)
12. Sarcasm	3.38 (1.11)	3.24 (1.06)	3.78 (1.14)
13. Cynicism	3.63 (1.24)	3.47 (1.17)	4.05 (1.31)

### Relationship Between Demographics, Humor, Depression, Anxiety, and Stress

Inter-scale correlations between all the variables were provided in Table 2.

**Table 2:** Full Correlation Matrix of the Collected Variables

Variable	1	2	3	4	5	6	7	8	9	10	11	12
1. Age											
2. Gender	-.12**											
3. Depression	-.16***	.01										
4. Anxiety	-.17***	.11**	.61***									
5. Stress	-.19***	.04	.76***	.64***								
6. Fun	-.04	-.11**	.04	.05	.05							
7. Humor	.11**	.09*	-.12**	-.11**	-.10**	.39***						
8. Nonsense	.10**	-.16***	.10**	.02	.07	.43***	.46***					
9. Wit	.06	-.11**	-.04	-.08*	-.01	.42***	.55***	.36***				
10. Irony	-.02	-.10**	.16***	.09*	.15***	.46***	.36***	.40***	.51***			
11. Satire	.14***	-.18***	.01	-.03	.02	.38***	.48***	.35***	.51***	.55***		
12. Sarcasm	-.03	-.20***	.08*	.01	.07	.37***	.24***	.29***	.44***	.60***	.55***	
13. Cynicism	-.01	-.21***	.17***	.06	.12***	.30***	.23***	.40***	.33***	.57***	.54***	.61***

*Note*. *N* = 686.

*p < .05. **p < .01. ***p < .001.

Since both age and gender appeared to affect both CSM and DASS-21 scores, the relationship between these two scales was analyzed controlling for age and gender through partial correlation analysis, as shown in Table 3. Our sample showed specific differences with reference to the relationships among the variables.

**Table 3:** Partial Correlations Among Variables Controlled for Age and Gender

Variable	Fun	Humor	Nonsense	Wit	Irony	Satire	Sarcasm	Cynicism
Depression	.04	-.11**	.12**	-.03	.16***	.02	.07	.17***
Anxiety	.06	-.09*	.05	-.07	.10*	.01	.03	.08*
Stress	.05	-.08*	.10*	.00	.16***	.05	.07	.13**

*Note*. *N* = 686.

*p < .05. **p < .01. ***p < .001.

Table 3 shows how two lighter styles and two dark styles reported significant correlations that can be deemed as relatively small in terms of effect size. Specifically, benevolent humor was generally negatively correlated to depression, anxiety, and stress, while nonsense was positively related to depression and stress. Conversely, in consideration of the dark styles, both irony and cynicism demonstrated positive correlations with depression, anxiety, and stress.

### Predictors of Negative Emotional States

To test how much variance within each dimension of the DASS is explained by the comic styles, a hierarchical regression of the comic styles with depression, anxiety, and stress as components of the criteria (controlled for age and gender) was employed. Statistical assumptions for the model, such as homoscedasticity, multivariate normality, and absence of multicollinearity, were all met. The general results of the hierarchical linear regressions analyses are summarized in Table 4.

**Table 4:** Hierarchical Regression of the Comic Styles With DASS, Controlled for Age and Gender

Variable	Fun	Humor	Nonsense	Wit	Irony	Satire	Sarcasm	Cynicism	Δ*R*^2^
Depression	-0.002	-0.20***	0.13**	0.06	0.20	-0.04	0.06	0.13*	0.09***
Anxiety	0.07	-0.14**	0.06	-0.11*	0.15**	0.01	-0.06	0.04	0.04***
Stress	0.01	-0.18***	0.10*	-0.03	0.18**	0.02	-0.05	0.06	0.08***

*Note*. *N* = 686.

*p < .05. **p < .01. ***p < .001.

The regression analyses demonstrated that the lighter and darker styles were differently related to the three facets of emotional distress. Overall, benevolent humor was the only style of humor related to all three constructs, while varying relationships emerged in other categories of humor. Depression was negatively predicted by benevolent humor and positively predicted by nonsense and cynicism (9% explained variance); anxiety was positively predicted by irony, while negatively predicted by benevolent humor and wit (4% explained variance); stress was best predicted by irony, cynicism, and nonsense, while negatively predicted by benevolent humor (8% explained variance).

## Discussion

The current study investigated how specific categories of humor related to three facets of emotional distress, namely depression, anxiety, and stress. Overall, the results of this study showed that the eight comic styles related differently to these facets. The findings of the present research propose negative links between the habitual use of benign humor and emotional distress while nonsense, irony, and cynicism were primarily associated with emotional distress. When considering the lighter styles, benevolent humor showed the strongest (negative) correlations with the three dimensions considered, while nonsense was a positive predictor of depression and stress. With reference to darker styles, irony was found to be unrelated to depression but was observed as the best predictor of anxiety and stress. Further, cynicism was the only (positive) predictor of depression, and no relationships emerged with regard to satire or sarcasm. Clinical depressed people were found to use self-defeating humor more and self-enhancing humor less than non-depressed (Kfrerer et al., 2019). Cynicism is aimed at devaluing commonly recognized values and cynics exhibit a negative and destructive attitude, that can be reflected in poor well-being by lowering the quality of relationships (Ruch, Wagner, & Heintz, 2018). It can be assumed that depressed people, having a negative view of self, may prove envy and frustration towards people, that are often associated with depression (Leahy, 2021). Cynicism may become a way to directly attack others and express their discomfort.

The main finding that emerged from this study is the negative relationship between benevolent humor and depression, anxiety, and stress, a finding that aligns with existing literature, which confirms the negative relationship of this style of humor with emotional distress. Previous studies have established links between good-natured humor and more effective emotion regulation (Samson & Gross, 2012). Moreover, individuals who habitually use benign humor tend to produce a greater share of positive reinterpretation, thereby resulting in minor stressful, anxious, and depressive experiences in their day-to-day life lives (Perchtold et al., 2019). Moreover, social and interpersonal aspects related to benevolent humor may influence health and well-being by increasing one's level of social support (Martin, 2019). As the intent of benevolent humor is to arouse sympathies toward the shortcomings of fellow humans by discovering discrepancies in everyday experiences and treating them with humor and benevolence, it represents an effective strategy for coping with stressful experiences (Lefcourt & Martin, 2012). Thus, benevolent humor can help one understand the incongruities of life and the imperfections of the world, enabling them to treat others benevolently, encouraging the reappraisal of negative situations, and consequently, decreasing negative emotions and improving well-being (Dionigi et al., 2021; Perchtold et al., 2019). Further, recent findings reveal that benevolent humor is negatively related to neuroticism (Dionigi et al., 2022; Ruch, Wagner, & Heintz, 2018), which, in turn, is related to depression, anxiety, and stress (Friedman, 2019). Neuroticism is an important risk factor for psychiatric traits such as depression (Kendler & Myers, 2010; Klein et al., 2009), and is longitudinally associated with episodic life stress (Schneider, 2004), which represents a major risk factor for anxiety symptoms and disorders (Clark et al., 1994).

Nonsense was found to be positively related to depression and stress, which may be explained by the unresolved incongruity within nonsense, which relates to experimentation with strangeness and ridiculousness with no specific purpose (Ruch & Heintz, 2016). Many studies have linked rumination, defined as repetitive thoughts about emotionally relevant experiences, with mood disorders, particularly exacerbated stress and depression (Whisman et al., 2020). Ruminative thinking has also been linked to distorted interpretations of life events and augmented pessimism concerning positive events in the future (Lyubomirsky & Nolen-Hoeksema, 1995). People who enjoy nonsense allow their minds to play with sense and nonsense without the need for resolving such incongruities (Ruch, Heintz, et al., 2018), and thus, may be more vulnerable to harboring repetitive thoughts regarding the negative aspects of their lives, thereby causing higher psychological distress.

Irony was found to be the best predictor of anxiety and stress. This comic style reflects a contrast or incongruity between expectations for a situation and its actuality, intending the opposite of the expressed meaning. Similar to anxiety and stress, irony relates to emotional experience and leads to emotional responses. Anxiety is a subjective feeling of fear or tension that leads to the perception of diverse and objectively harmless situations as threatening, causing individuals to react inappropriately to them (Spielberger, 1966). The association between anxiety and cognitive distortions in the face of emotionally ambiguous situations is well-proven, as anxious individuals frequently interpret and evaluate such contexts more negatively, thereby intensifying their anxiety (Hayes & Hirsch, 2007). Due to the ambiguity involved in irony, it is capable of conveying stressful stimuli for some people (Milanowicz et al., 2017). Additionally, worrying can direct cognitive resources in a specific direction, without posing attention to other important information and irony may lead to a more ambiguous perception of events, thereby cognitively orienting the individual to select only the negative aspects of the events and ignore the situational factors that could otherwise have altered their negative interpretations. Irony may also be explained as a maladaptive coping strategy that individuals used to be perceived by others as capable of dealing with unpleasant and stressful situations while maintaining a high level of anxiety and stress. In this regard, existing empirical research has determined that coping with humor is positively related to worry, the cognitive aspect of anxiety (Davey, 1993), further demonstrating that the functions of worry are frequently associated with active cognitive coping while anxiety is linked to avoidant coping. Irony, as expressed in interactions, aims to create a mutual sense of superiority among others by stating things different from their intended meaning. Since depression is characterized by prolonged depressed feelings for most of the day, markedly diminished interest or pleasure in all, or almost all, activities, feelings of worthlessness, or excessive or inappropriate guilt nearly every day (APA, 2013), it seems reasonable that depression and irony.

While cynicism was unrelated to anxiety and stress, it was a positive predictor of depression. This style of humor aims to devalue commonly recognized values, characterizing a negative and destructive attitude. Cynics use disillusionment and mockery to highlight weaknesses in the world. Previous studies showed how cynicism was positively correlated to neuroticism (Dionigi et al., 2021; Ruch, Wagner, & Heintz, 2018), which, in turn, relates to anxiety and negative affectivity (Friedman, 2019). Furthermore, the use of dark humor, namely cynicism, sarcasm, and irony has been associated with antagonism (Perchtold et al., 2019), which represents the maladaptive extension of low levels of the classic “Big Five” trait, agreeableness (Suzuki et al., 2017). Using mockery to highlight weaknesses in the world may lead to a negative influence on well-being by lowering the quality of relationships (Ruch, Wagner, & Heintz, 2018). Moreover, as cynicism was positively related to worry (Dionigi et al., 2022), we expected a positive relationship across all three constructs. The missing link between cynicism, stress, and anxiety in this research may have occurred as a result of the instrument used to assess emotional distress. The Depression Anxiety Stress Scale (DASS-21) evaluates anxiety and stress involving items focused more on physical, rather than, psychological aspects (e.g., “I was aware of dryness of my mouth” and “I experienced trembling” (e.g., in the hands). The few research works conducted thus far using the Comic Style Markers highlighted the negative relationship between the mockery style, particularly cynicism, and cognitive facets of anxiety, such as worry (Dionigi et al., 2022) and cognitive reappraisal (Perchtold et al., 2019). Thus, we assume that the Comic Style Markers may be more centered on capturing psychological, rather than physical, aspects.

It is worth mentioning that the correlations reported in the study are relatively small. At the time of writing there is little research conducted using the Comic Styles Model, therefore a comparison with other studies is limited. However, previous studies that investigated the relationship between the comic styles and subjective well-being (Ruch, Wagner, & Heintz, 2018) and comic styles and worry (the cognitive component of anxiety) and well-being (Dionigi et al., 2021) found similar pattern of correlations. Moreover, previous studies showed how personality traits represent the best predictors of psychological well-being while humor styles added only a small portion of explained variance (Jovanovic, 2011). Again, Páez et al. (2013) investigated the incremental validity of humor styles in predicting happiness and psychological well-being, showing that, after controlling for personality traits, only two humor styles (self-enhancing and self-defeating) remained significant predictors of the two variables, adding only 2% of explained variance to the model. These results showed that the role of humor in predicting psychological well-being is limited (Ruch & Heintz, 2013), therefore we can state that our findings are in line with previous results.

Although these findings highlight a subject that has yet not been investigated, certain limitations regarding the findings require acknowledgment. First, the study was conducted on a sample of Italian participants, and hence, further research is essential to confirm these results for other cultures and nationalities. Second, only self-reports were employed. Another limitation of our study is that the increase in the familywise error rate was not controlled for in the reported statistical analyses. Overall, we consider our findings to be relatively preliminary and encourage replication. Future research should extend the assessment to multimethod data, such as other-reports to extend forms of obtained data beyond self-perceptions. Third, due to the correlational nature of the study, implications of causality cannot be established. Finally, the relationship between humor and emotional distress may be bidirectional. Other research designs are required to investigate the relationships of these variables even further.

In conclusion, the present study first investigated the relationships between the eight comic styles, and the three facets of emotional distress, namely depression, anxiety, and stress. Benevolent humor was negatively related to all three constructs and was the best (negative) predictor of depression. Irony was positively related to anxiety, which was best predicted by stress, while cynicism was found to relate positively to depression. These results support the proposition that specific categories of humor may affect emotional distress in various ways.

## Data Availability

The datasets generated during and/or analyzed during the current study are available from the corresponding author on reasonable request.

## Appendix

The eight comic styles can be differentiated as lighter or darker styles of humor, which collectively include *fun, humor, nonsense, wit, irony, satire, sarcasm, and cynicism* (Ruch, Heintz, et al., 2018). These styles were originally developed to describe literary work and are used here to describe individual differences. Specifically, the features are intention/goal (examples taken from wit: to illuminate like a flashlight; desire for being brilliant), object (words and thoughts), attitude of the agent (tense, vain, takes oneself seriously), behavior towards other people (callous, malicious; without sympathy for “victims”), ideal audience (educated society that appreciates wit), method (surprising punch line; “sensation” of the unusual combination), and linguistic peculiarities (brief, pointed, enjoying contrasting stylistic devices) (Ruch, Wagner, & Heintz, 2018).

The four lighter styles, which relate to benign and social affect, behaviors, cognitions, and goals are:

1. *Fun*: aimed at spreading good mood and good companionship. People using this comic style are considered to be social, jovial, and also agreeable.
2. *Humor*: aimed at arousing sympathy towards the shortcomings of a fellow human, discovering discrepancies in everyday experiences, and treating them in a humorous and benevolent manner.
3. *Nonsense*: based on experimentation with incongruities and ridiculousness with no specific purpose. It is mainly based on presenting ridiculous aspects of things that play with sense and logic, enjoying contradictions and absurdities.
4. *Wit:* which relates to the ability to create clever connections between ideas and thoughts, that are not necessarily connected.

Conversely, the darker styles These are generally negative styles of humor that lack this benevolent affect are mostly based on mockery and ridicule. Darker styles of humor include:

1. *Irony:* reflecting a contrast or incongruity between expectations for a situation and reality, characterizing the opposite of the expressed meaning. It is often used to state things in opposition to their meaning, thus confusing those who are not “part of the group”.
2. *Satire:* directed at criticizing and correcting shortcomings, misconduct, and moral wrongdoings with the intent to improve the world. Although it appears similar to sarcasm and cynicism in its criticism and ridicule, it is combined with the moral aim of correcting others.
3. *Sarcasm:* grounded on the need to be critical of others and convey contempt. Sarcasm can be used to criticize and ridicule others people, institutions or topics.
4. *Cynicism*: aimed at devaluing commonly recognized values, ridiculing the weaknesses in the world and disdaining moral concepts, which are considered ridiculous.
