# Correction of Alberto Dionigi, Mirko Duradoni, & Laura Vagnoli (2023). Understanding the Association Between Humor and Emotional Distress: The Role of Light and Dark Humor in Predicting Depression, Anxiety, and Stress

**DOI:** 10.5964/ejop.14059

**Published:** 2024-02-29

The initial published version of the article

DionigiA.
DuradoniM.
VagnoliL.

(2023). Understanding the association between humor and emotional distress: The role of light and dark humor in predicting depression, anxiety, and stress.
Europe's Journal of Psychology, 19(4), 358370
10.5964/ejop.10013

was produced with an incorrect issue number. The corrected citation, which changes the issue number from 3 to 4, is shown above. The publisher apologizes for the error and any inconvenience caused.

